# Relationships Among Patients' Interpersonal Behaviors in Sessions, Therapist Competence, and the Therapeutic Alliance in Cognitive Behavior Therapy: A Cross‐Lagged Panel Analysis

**DOI:** 10.1002/jclp.70040

**Published:** 2025-08-30

**Authors:** Ulrike Maaß, Michael Witthöft, Yvonne Marie Junga, Daniela Hahn, Florian Weck

**Affiliations:** ^1^ Department of Clinical Psychology and Psychotherapy University of Potsdam Potsdam Germany; ^2^ Department of Clinical Psychology and Psychotherapy Ruhr‐University Bochum Bochum Germany; ^3^ Clinical Psychology, Psychotherapy and Experimental Psychopathology University of Mainz Mainz Germany

**Keywords:** competence feedback, interpersonal behavior, major depression, therapeutic alliance, therapeutic competencies, therapist bias

## Abstract

**Objective:**

To investigate the reciprocal relationships between patient interpersonal behavior (IB), therapist competence, and the alliance within‐ and between‐persons.

**Methods:**

A secondary analysis was conducted of a randomized controlled trial with 67 cognitive behavior therapists and 114 patients with depression. The therapists evaluated their patients' IB, and they both judged the alliance. Pre‐ and post‐treatment, the patients indicated their general levels of interpersonal problems. Every fourth session, independent raters judged the patients' IB and therapists' competence. Random‐intercept cross‐lagged panel analyses were calculated.

**Results:**

First, intraindividual deviations in patient IB were associated with deviations in therapist competence or the alliance (within‐person); and patients with generally more positive IB experienced not only better alliances but also more competent therapists (between‐person). Second, the perspective of the evaluating person and the time interval were decisive, as significant cross‐lagged effects between patients' IB in one session and the alliance in subsequent sessions (and vice versa) only occurred from the therapist perspective, but not when the patient perspective was considered. Third, patients with more interpersonal problems before treatment did not show more negative IB, nor did they experience poorer alliances or less competent therapists.

**Conclusion:**

The study supports the idea that patient IB is as an important part of both therapeutic competence and the alliance. However, therapists and raters may be prone to evaluation bias, interpreting negative patient IB as a sign of poor alliance or competence (and vice versa). Finally, the extent of interpersonal problems before treatment does not substantially influence the three process variables.

**Trial Registration:** https://clinicaltrials.gov/study/NCT02479594.

## 1 Introduction

Psychotherapy is a dyadic process that requires close cooperation between the therapist and patient. Accordingly, both sides are responsible for creating an atmosphere of trust. While therapists can positively influence this collaboration with their competence and their ability to establish a sustainable alliance, patients influence the relationship largely through their interpersonal behavior (IB) in sessions. There is evidence that, for example, patients respond to therapists' hostile‐controlling behavior with greater hostility, which in turn is associated with poorer therapy outcomes (Henry et al. 1986). Furthermore, certain patient IBs are particularly related to worse treatment outcomes, such as resistance, submissiveness, and hostility toward the therapist (e.g., Constantino et al. 2021). In contrast, positive correlations with therapy success have been found for patient engagement, self‐disclosure, emotional expression, positive expectations, and a secure attachment style (Constantino et al. 2021; Coyne et al. 2019).

Although cross‐sectional studies indicate a close relationship between patient IB and the alliance (e.g., Huber et al. 2019), we have relatively little longitudinal data on the relationships with therapeutic competence. In addition, the relationships within and across therapy sessions have not yet been sufficiently illuminated. There are still some unanswered questions, such as: Are changes in therapeutic competence or the alliance associated with changes in patient IB, and vice versa? Do such changes in one session also predict changes in behavior in subsequent sessions? The present study aims to examine such reciprocal associations between patient IB, therapist competence, and the therapeutic alliance during therapy.

### 1.1 Patients' Interpersonal Problems and Behaviors

While therapist competence is usually described in terms of the therapist's skill level in delivering treatment (Waltz et al. 1993), the therapeutic alliance determines the quality of the relationship between the therapist and the patient, including the emotional bond and mutual agreement on tasks and goals in therapy (Bordin 1983). Patient IB is often seen as part of the therapeutic alliance (Richtberg et al. 2016), but beyond that, it is not uniformly conceptualized. Some authors focused primarily on individual negative behaviors such as resistance (e.g., Westra et al. 2012), while others examined the general pattern of interpersonal and intrapsychic perceptions along dimensions of circumplex models, such as agency versus communion (Interpersonal Circumplex Model; Horowitz et al. 2000) or affiliation and interdependence (Structural Analysis of Social Behavior Model; Henry et al. 1986). However, Richtberg et al. (2016) point out that many measurements based on these approaches are often time‐consuming and complex. The authors have therefore developed an observer‐based measurement that can be applied more quickly. More precisely, the authors describe patient IB as the patient's observable contribution to the therapeutic process, including positive and negative behaviors within single therapy sessions. Their own Assessment Form of Patient IB (AFPIB; Richtberg et al. 2016) assesses the most studied patient IBs, including several positive IBs (e.g., reporting openly of one's own accord, being attentive, being proactive, and being friendly to the therapist) and negative IBs (e.g., degrading the therapist, expressing doubts about the treatment concept, and seeming distanced from the therapy process). In a study of patients suffering from hypochondriasis, scores on the AFPIB were able to predict therapeutic success and explain up to 13% of the variance in outcomes (Richtberg et al. 2017).

### 1.2 Correlations Between Patient IB, Therapeutic Competence, and the Alliance

Neither patient IB, therapist competence, nor the alliance are assessed in isolation from the interaction between both parties, as all behavior invites a reaction (McFarquhar et al. 2018).

Although there are a few studies that have explicitly examined the interaction of these variables in longitudinal designs, the results are limited in their comparability because the studies differ in terms of the measurement of patient IB, the time intervals examined, and the assessment perspectives (e.g., self‐report vs. independent rater). For example, an early study on interpersonal psychotherapy with 35 depressive patients indicates a strong connection between patient IB and therapist competence (Foley et al. 1987). The authors reported that greater supervisor‐rated patient difficulty (e.g., hostility, impatience, and/or defensiveness) within seven sessions was associated with lower self‐ and supervisor‐rated therapist competence during treatment. However, the authors focused primarily on negative patient behavior and pointed out that the assessment of therapists' performance was not independent of the “difficulty” of the patients. Notably, another study that used the AFBIP to record both negative and positive behaviors did not find consistent relationships with therapist competence and the alliance (Weck et al. 2016). The authors examined the associations between patient IB in session 1 and the alliance and competence measured in session 6 during cognitive behavior therapy (CBT) with 85 patients with agoraphobia and panic disorder. Although patient IB was positively associated with an observer‐based evaluation of the therapeutic alliance, it was not significantly correlated with therapist competence. However, the study's conclusions are somewhat limited because only two measurement points with a relatively long time interval (6 weeks) were examined.

In contrast, a recent longitudinal study examined more closely the interplay of variables throughout the whole process of therapy. Huber et al. (2021) applied random‐intercept dynamic panel models in a sample of 386 patients who were diagnosed with affective disorders and received psychodynamic psychotherapy. The patients answered questionnaires every 5th session regarding the alliance and their agentic behavior within therapy (e.g., engagement, acceptance of responsibility, and implementation of ideas). The results showed that an increase in patient agentic behavior predicted a decrease in alliance during sessions 1–20, with this effect decreasing over time. At the same time, higher alliance levels at baseline were associated with later increases in agentic behavior. The authors concluded that a solid alliance at the beginning of therapy is a precondition for patients′ ability to behave with greater agency and that further gains in accountability are necessarily accompanied by diminished therapeutic alliances. Therapists who fail to address agency in their interventions may risk ruptures in the therapeutic alliance.

#### 1.2.1 Trait‐ and State‐Like Components

In addition, Huber et al. (2021) differentiated between trait‐like and state‐like components of patient IB and the alliance. While the trait‐like component of a variable reflects relatively stable individual differences across time and situations, the state‐like component manifests in specific situations as intraindividual deviations from the trait level. Considering this distinction, Huber et al. (2021) reported that when controlling for the general level of agency (trait‐like), the individual increase in agency (state‐like) was still associated with subsequent symptom improvement. However, these findings are based on patient self‐reports and therefore cannot make any statements about the actual behavior exhibited in a therapeutic session.

Yet, the distinction between self‐ and observer‐based reports is important, as therapists tend to overestimate their own competences in comparison with the perspective of independent raters (Caron et al. 2020). Similarly, therapists and patients are known to have different perspectives on their alliance as a whole and/or individual aspects of it (Shick Tryon et al. 2007). The study by Huber et al. (2021) had long time intervals between measurement points (every 5 sessions), potentially missing microprocesses between patient IB and a change in the therapeutic alliance in response to it (or vice versa).

#### 1.2.2 The Role of Interpersonal Problems

Although there are many other longitudinal studies that have examined the therapeutic alliance and differentiated between trait‐like and state‐like components in the process of therapy (e.g., Falkenström et al. 2016; Flückiger et al. 2020; Zilcha‐Mano and Fisher 2022), the alliance was rarely linked to patients' concrete in‐session behavior. Instead, the alliance has been studied primarily in connection with general severe interpersonal problems (e.g., Coyne et al. 2019). Patients' general level of interpersonal problems is conceptualized as a relatively stable trait, defined as “social difficulties and trouble creating or maintaining healthy relationships with others” (Harris et al. 2023, p. 303). It can be assumed that such patient traits also influence their IB in sessions (Dermody et al. 2016). For this reason, it seems advisable to also consider the general level of interpersonal problems when examining the interactions between patient IBs in sessions and therapist competence or alliances.

### 1.3 Summary

In summary, the evidence regarding the interplay between patient IB, therapist competence, and the therapeutic alliance in the course of therapy indicate a mutual connection between the variables. That means that changes in one variable should also result in changes in the other variable in the therapy process. In addition, it can be assumed that a higher level of interpersonal problems has a rather negative impact on patient IB, therapist competence, and the therapeutic alliance. However, the comparability of the research to date is limited because studies differ greatly in terms of the assessment perspectives (therapist, supervisor, independent rater) and the variables examined (interpersonal problems vs. in‐session behavior; negative vs. positive in‐session behavior). In addition, many studies usually examined time intervals of several weeks, which means that short‐term interactions in the sense of action‐reaction could not yet be detected. Against this background, there is a need for longitudinal studies that take these factors into account and thus examine the relationships in a more nuanced way.

### 1.4 This Study

The present study aimed to answer the question whether changes in therapeutic competence or the alliance would be reciprocally associated with changes in patient IB. In addition, we investigated whether intraindividual changes in one session would also predict changes in behavior in subsequent sessions. Finally, we explored the role of patients' level of interpersonal problems. Based on the literature described above, our hypotheses were:

H1: Positive patient IB is significantly correlated with better therapist competence and better therapeutic alliances both within‐ and between‐persons.

H2: In the course of therapy, there are significant cross‐lagged effects. That is, positive patient IB in one session is significantly associated with better therapist competence and better therapeutic alliances in subsequent sessions, and vice versa.

H3: Patients with more interpersonal problems before therapy show more negative patient IB in sessions, experience therapists with lower competences, and have lower alliances.

In addition, we considered (a) multiple perspectives on patient IB (either judged by independent raters or therapists) and the alliance (either judged by therapists or patients), and (b) two time intervals for nuanced insights into the relationships (i.e., a microperspective with data from each session and a mesoperspective with data from every 4th session).

## 2 Materials and Methods

This study is a secondary analysis of a randomized controlled trial (RCT) that compared the effects of competence feedback and no competence feedback on the therapeutic skills of psychotherapy trainees and patient outcomes (Weck et al. 2021). Patients with a diagnosis of major depression received 20 weekly individual CBT sessions (50 min), which adhered to a German protocol for CBT (Hautzinger 2013) that incorporates elements of cognitive therapy (e.g., changes in dysfunctional cognitions or training of interpersonal skills; Beck, Steer, et al. 1996; Beck, Rush, et al. 1996) and behavioral activation (Jacobson et al. 1996). The treatment was conducted at the outpatient unit of the University of Mainz (Germany). Every fourth therapy session, the therapists received supervision from licensed supervisors. The competence feedback in this study was based on the items of the Cognitive Therapy Scale (CTS; Weck et al. 2010), which were evaluated by independent raters. The therapists received quantitative feedback on their improvement in the CTS as well as brief qualitative suggestions for further improvement (see the original study for detailed information). The authors reported that while competence feedback (vs. no feedback) improved therapist competence, it was not associated with more depression reduction or better alliances. Ethical approval for this study was obtained from the Institutional Review Board of the University of Potsdam (Germany; No. 31/2016), and the study protocol was registered with www.ClinicalTrials.gov (NCT02479594).

### 2.1 Sample

Between August 2015 and April 2020, participants were recruited at the outpatient unit of the University of Mainz and screened for diagnoses via the German versions of the Structured Clinical Interview for DSM‐IV (First et al. 1997), the Beck Depression Inventory‐II (Beck, Steer, et al. 1996; Beck, Rush, et al. 1996), and the Hamilton Rating Scale for Depression (Hamilton 1960). The inclusion criteria were (a) age ≥ 18 years, (b) major depression status, (c) BDI‐II score ≥ 20 and HRSD score ≥ 14, (d) fluency in German, and (d) written informed consent. The exclusion criteria were acute suicidality, substance dependence, schizophrenia, schizoaffective disorder, bipolar disorder, cluster A or B personality disorder, or concurrent psychotherapy. Agreement between the diagnosticians and a trained expert was satisfactory (κ = 0.79; *p* < 0.001).

#### 2.1.1 Patients, Therapists and Raters

The sample included 114 patients (64 women, 56.14%; mean age *M* = 40.26 years, SD = 14.29, range 18–74). Ninety‐one participants (79.82%) were diagnosed with recurrent depression, 70 (61.40%) with at least one additional disorder (i.e., an anxiety, somatoform, or personality disorder), and 40 (35.09%) took prescribed antidepressants. Sixty‐one participants (53.51%) had at least 12 years of education, and 67 (58.77%) were married or cohabiting.

Sixty‐seven clinical psychologists (M.Sc. degree) who were in training to become licensed cognitive behavior therapists participated in the present study (57 women, 85.07%; mean age *M* = 29.25 years, SD = 3.97, range 25–49; clinical experience *M* = 2.50 years, SD = 0.92). The therapists received 16 h of training in CBT for major depression and supervision approximately every fourth therapy session (i.e., the competence‐feedback group [CFG] received supervision in addition to written competence feedback).

Eleven licensed clinical psychologists (six with PhDs) served as raters and feedback providers (clinical experience *M* = 6.56, SD = 2.03; range: 3–9). In preparation for the study, the raters were trained (12 h) in using the competence scales and providing feedback. During the study, they participated in 14 additional training sessions to avoid rater drift. The raters were not blinded to the group allocation (i.e., competence feedback vs. no such feedback).

### 2.2 Study Design and Randomization

The therapists (*n* = 67) were randomly assigned to either the CFG or the control group (CG) at an allocation ratio of 1:1 by an independent scientist via the website www.randomizer.org. The patients (*n* = 114) were randomly assigned to 45 therapists, with most therapists treating multiple patients. Therapists in the CFG received feedback after sessions 1, 5, 9, 13, and 17, while those in the CG received feedback after session 20.

### 2.3 Measurements

#### 2.3.1 Patient IB

The therapists evaluated their patients' IBs each session, and the independent rater evaluated the patients' IBs every fourth session (sessions 1, 5, 9, 13, and 17) with the AFPIB (Richtberg et al. 2016). The AFPIB is an observer‐based 10‐item questionnaire with a 5‐point rating scale (0 = examples of negative patient IB, 4 = examples of positive patient IB). An example item is “*Showing interest (*vs*. not showing interest)*. The patient shows his or her interest in the content of the therapy session.” The extreme points of the corresponding rating scale are “0 = The patient shows no interest in absorbing the contents of the therapy session; e.g., she or he does not ask questions,” and “4 = The patient is very interested in absorbing the content of the therapy session; for example, she or he asks in‐depth questions.” The internal consistency at session 1 was good: therapists: *α* = 0.72, Rater 1: *α* = 0.75, and Rater 2: *α* = 0.75. Rater agreement was fair to excellent (Cicchetti 1994): ICC_(1,2)_ = 0.51 (session 1), ICC_(1,2)_ = 0.67 (session 5), ICC_(1,2)_ = 0.72 (session 9), ICC_(1,2)_ = 0.78 (session 13), ICC_(1,2)_ = 0.63 (session 17), all *p*s < 0.001.

#### 2.3.2 Therapist Competence

The raters evaluated the therapists' competencies every fourth session with the German version of the CTS (Weck et al. 2010). This scale consists of 14 items assessing therapist competence in CBT, in areas such as “agenda setting,” “guided discovery,” and “resource activation” (rating scale: 0 = poor to 6 = excellent). The interrater reliability for the CTS mean scores between independent raters was fair to good (Cicchetti 1994): ICC_(1,2)_ = 0.62 (session 1), ICC_(1,2)_ = 0.74 (session 5), ICC_(1,2)_ = 0.65 (session 9), ICC_(1,2)_ = 0.51 (session 13), ICC_(1,2)_ = 0.71 (session 17), all *p*s < 0.001.

#### 2.3.3 Therapeutic Alliance

Each session, the therapists and patients completed the German Helping Alliance Questionnaire (HAQ; Bassler et al. 1995) to assess the collaborative and affective bond between the therapist and patient with 11 items and a 6‐point rating scale (1 = strongly disagree to 6 = strongly agree; example item: “I feel the patient is working together with the therapist in a joint effort.” The internal consistency at session 1 was good: *α* = 0.79–0.82 (therapists, patients).

#### 2.3.4 Patient Interpersonal Problems

The patients indicated their level of interpersonal problems before and after treatment via the German version of the 64‐item Inventory of Interpersonal Problems (IIP; Horowitz et al. 2000). The IIP is a self‐report measure of things people find too difficult, such as making friends, and things they feel they do too often, such as losing their temper (rating scale: 0 = not at all to 4 = extremely). An example item is “I am too aggressive toward other people.” The internal consistencies were good (pre: *α* = 0.92, post: *α* = 0.95).

### 2.4 Data Analysis

The analyses were performed via RStudio 1.1.456 (RStudio Team 2015) and the lavaan package (Rosseel 2012). We determined descriptive statistics per study group (competence feedback vs. no feedback) for all the variables. We employed random‐intercept cross‐lagged panel models (RI‐CLPM; Hamaker et al. 2015) to analyze the reciprocal relationships among our variables over time. This allowed us to examine the predictive power of different variables and gain insights into causal relationships and their direction. RI‐CLPMs also distinguish between state‐like (e.g., session‐to‐session changes) and trait‐like dynamics (e.g., stability from session to session). Interested readers should refer to Mulder and Hamaker (2021) for a detailed description of the method (including sample analysis codes). Our analysis codes can be found with the Open Science Framework (https://doi.org/10.17605/OSF.IO/69CMQ).

#### 2.4.1 RI‐CLPMs

The RI–CLPM extends the traditional cross‐lagged panel model for analyzing longitudinal data with a multilevel structure, allowing for the observation of relationships at each time point and across time. For example, each patient in this study provided different therapeutic alliance ratings across sessions. These ratings represent changes within a patient over time, also referred to as the within‐person level. Additionally, each patient exhibited overall patterns that differed from those of other patients, for example, because they were in different study groups or had a generally more positive view of the alliance. These ratings are also referred to as the between‐person level. The RI‐CLPM separates the patient's responses into “trait‐like” differences, which are stable over time, and “state‐like” differences, which vary from session to session. The *trait‐like* components are represented by random intercepts that measure between‐person differences. For example, some patients might generally show more positive behaviors than other patients do, which might be associated with generally better therapeutic alliances. In contrast, the *state‐like* components are represented by cross‐lagged and autoregressive parameters that measure within‐person differences over time. For example, a positive *cross‐lagged parameter* indicates that intraindividual deviations in alliance in session 1 predict deviations in patient IB in session 2. Similarly, a positive *autoregressive parameter* indicates whether a change in a person's average alliance ratings from session 1 carries over into session 2.

We also controlled for study group membership (competence feedback vs. no feedback), patients' pre‐treatment interpersonal problems, and post‐treatment interpersonal problems. Therefore, random intercepts of patient IB, therapist competence, and the alliance were regressed on these control variables. Supporting Information S1: Material 1 displays an example RI‐CLPM for five sessions.

H1 (positive correlations between patient IB and therapist competence and between patient IB and the alliance) was examined by the correlations at the within‐ and between‐person levels. H2 (positive cross‐lagged effects between patient IB and therapist competence and between patient IB and the alliance) was examined by interpreting the standardized cross‐lagged effects from one session to the next. H3 (negative correlations between patients' interpersonal problems before therapy and patient IB in sessions, therapist competencies, or the alliance) was examined by interpreting the correlations between IIP scores before treatment and the random intercepts of the process variables. The correlations were interpreted as small (0.10), medium (0.30), or large (0.50; Cohen 1988). Standardized cross‐lagged effects were interpreted as small (0.03), medium (0.07), or large (0.12; Orth et al. 2024).

We considered recommendations from the literature to ensure sufficient power in this secondary analysis. For example, Falkenström et al. (2022) demonstrated that the RI‐CLPM was superior to multilevel modeling in modeling cross‐lagged effects with small sample sizes (*n* = 50−200) and between 3 and 15 measurement points. In addition, Zyphur et al. (2020) noted that constraints increase statistical power. Therefore, we applied constraints in our analyses.

#### 2.4.2 Time Intervals and Perspectives of the Raters, Therapists, and Patients

We were interested in two different periods. First, we analyzed models with five measurement points on the basis of the session intervals of the raters (i.e., every fourth session: sessions 1, 5, 9, 13, and 17). This four‐session interval included four models depending on the perspectives of the rater, therapist, and patient: (1) the rater model, which focused on the raters' reports of the patients' IB and the therapists' competence; (2) the therapist model, which focused on the therapists' evaluations of their patients' IB and the therapeutic alliance; and (3) the patient–therapist model, which focused on the therapists' reports of their patients' IB and the patients' evaluation of the therapeutic alliance. Second, we analyzed models with all 20 measurement points at session‐to‐session intervals. These analyses included only two models: (1) the therapist model and (2) the patient–therapist model.

#### 2.4.3 Model Selection Process

Before interpreting the results, the RI‐CLPM models were built step by step to test different model assumptions (constraints, detrending, and residual vs. observational approaches). In Step 1, we built a basic model for each model in the *session‐to‐session interval* and the *four‐session interval*. We then tested step by step whether certain paths could be constrained over time: the cross‐lagged paths, autoregressive paths, variances and covariances. In Step 2, we tested for time trends, which can bias the cross‐lagged effects (Falkenström et al. 2023). Thus, we compared the best fitting model from Step 1, which estimated separate intercepts for each time point (i.e., a detrended model in which time trends were removed), with a model that constrained the intercepts to be equal over time (i.e., a nondetrended model). In Step 3, we compared the best fitting model from Step 2 with a “predetermined RI‐CPLM” to allow the initial deviations to correlate with the individual intercepts. This procedure followed an observational approach, as suggested by Andersen (2022), who critiques the RI–CLPM for using residuals to differentiate between within‐ and between‐person effects. The author argues that these effects can also be directly estimated using observed variables. A major advantage of doing so is that the observational model imposes less restrictive assumptions about the initiation of processes, especially in shorter panels with fewer repeated measures. Andersen suggests comparing the residual‐level model with and without the covariances between the initial deviations and the individual effects fixed to zero. Using chi‐square difference tests, model comparisons were based on the root mean square error of approximation (RMSEA < 0.08), the standardized root mean square residual (SRMR < 0.08), and the comparative fit index (CFI > 0.90; Byrne and Watkins 2003). Full information maximum likelihood estimation was used to address missing values.

## 3 Results

Table 1 displays descriptive statistics for all measures. The study groups did not significantly differ from each other, except for therapeutic competencies. The feedback group achieved significantly higher competence ratings than the CG (*d*s > 0.55).

**Table 1:** Descriptive statistics for all measurements.

Rater							
CTS				AFPIB			
	CFG	CG			CFG	CG	
	*M (SD)*	*M (SD)*	*d*		*M (SD)*	*M (SD)*	*d*
Session 1	3.47 (0.66)	3.36 (0.56)	0.18	Session 1	2.91 (0.42)	2.87 (0.37)	0.13
Session 5	3.79 (0.61)	3.26 (0.60)	**0.88**	Session 5	2.92 (0.52)	2.86 (0.41)	0.13
Session 9	3.72 (0.66)	3.25 (0.52)	**0.78**	Session 9	2.84 (0.51)	2.90 (0.57)	0.10
Session 14	3.69 (0.63)	3.37 (0.51)	**0.55**	Session 14	2.92 (0.56)	2.92 (0.51)	0.01
Session 17	3.79 (0.65)	3.25 (0.62)	**0.86**	Session 17	2.92 (0.53)	2.91 (0.50)	0.02

Therapist							
HAQ				AFPIB			
Session 1	4.37 (0.51)	4.34 (0.57)	0.05	Session 1	2.88 (0.45)	2.98 (0.49)	0.22
Session 2	4.50 (0.46)	4.53 (0.52)	0.07	Session 2	2.92 (0.52)	3.03 (0.58)	0.23
Session 3	4.47 (0.52)	4.49 (0.55)	0.05	Session 3	2.91 (0.53)	3.01 (0.61)	0.18
Session 4	4.57 (0.53)	4.53 (0.49)	0.09	Session 4	2.94 (0.55)	2.99 (0.62)	0.07
Session 5	4.59 (0.50)	4.57 (0.52)	0.03	Session 5	2.95 (0.54)	3.03 (0.64)	0.13

Patient							
HAQ				IIP			
Session 1	4.65 (0.57)	4.65 (0.54)	0.00	Pre‐treatment	1.62 (0.49)	1.57 (0.51)	0.11
Session 2	4.74 (0.57)	4.73 (0.52)	0.02	Post‐treatment	1.57 (0.57)	1.50 (0.57)	0.12
Session 3	4.80 (0.54)	4.67 (0.53)	0.26				
Session 4	4.87 (0.59)	4.78 (0.50)	0.17				
Session 5	4.90 (0.55)	4.76 (0.62)	0.23				

*Note*. Bolded effects are statistically significant at *p* ≤ 0.05.

Abbreviations: AFPIB = assessment form of patient interpersonal behavior; CFG = competence feedback group; CG = control group; CTS = cognitive therapy scale; Group = study group (competence vs. no feedback); HAQ = helping alliance questionnaire; IIP = interpersonal problems.

### 3.1 Model Building

**Figure 1:**
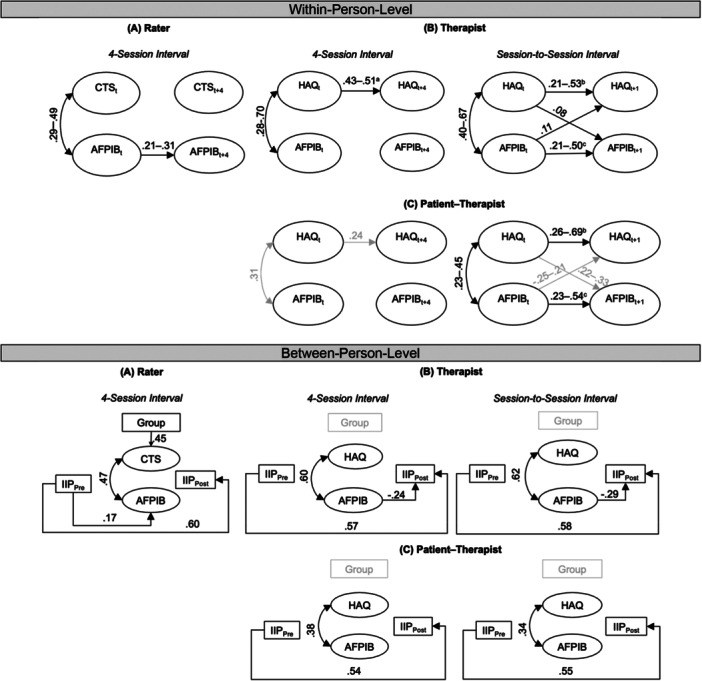
Schematic summary of the results based on the standardized beta coefficients from the RI‐CLPMs. Only significant effects with at least *p* ≤ 0.05 are displayed. gray coefficients = effects that were difficult to interpret, as they occurred only sporadically or without a clear pattern. ^a, b, c^Significant effects refer to sessions in (A) the middle and end phases; (B) the beginning and end phases; and (C) the beginning, middle, and end phases of therapy. (A) Rater. (B) Therapist. (C) Patient‐Therapist. AFPIB = assessment form of patient IB; CTS = cognitive therapy scale; Group = study group (competence vs. no feedback); HAQ = helping alliance questionnaire; IIP = interpersonal problems.

The results of the model selection process are displayed in Supporting Information S1: Materials 2 to 4. The observational approach had the best model fit for all the models with freely estimated intercepts to adjust for time trends (except in the rater model, where a nondetrended model fit best).^1^ The final models included different extents of constraints, ranging from no constraints (patient–therapist model in the session‐to‐session interval), constrained cross‐lagged effects only (both therapist models and patient–therapist model in the four‐session interval), and constrained autoregressions and cross‐lagged effects (rater model). Almost all the final models yielded good model fits, rater model (four‐session interval): CFI = 0.98, RMSEA = 0.05, SRMR = 0.06; therapist model (session‐to‐session interval): CFI = 0.91, RMSEA = 0.07, SRMR = 0.07; therapist model (four‐session interval): CFI = 0.99, RMSEA = 0.04, SRMR = 0.08; patient‐therapist model (four‐session interval): CFI = 0.96, RMSEA = 0.08, SRMR = 0.07. The model fit of the patient‒therapist model at the session‐to‐session interval was acceptable: CFI = 0.87, RMSEA = 0.08, SRMR = 0.09. The detailed results of each model are displayed in Supporting Information S1: Materials 5 to 7. A summary of the results from the RI‐CLPM models is displayed in Figure 1, which shows abbreviated and schematic RI‐CLPMs per perspective (rater, therapist, patient–therapist), time interval (each session vs. every 4th session), and level (within vs. between person).

Within‐and Between‐Person Correlations Between the Process Variables.

Overall, H1 was largely confirmed, as there were positive correlations in almost all models (Supporting Information S1: Materials 6 to 7). At the within‐person level, the results indicate that intraindividual deviations in patient IB correlated positively with intraindividual deviations in therapist competence according to the raters (*r*_*w*_ = 0.29–0.49; Figure 1, Supporting Information S1: Material 5) and in the alliance according to the therapists (*r*_*w*_ = 0.28–0.70; Figure 1, Supporting Information S1: Material 6). At the between‐person level, patients who generally behaved more positively also had more competent therapists according to the raters (*r*_*b*_ = 0.47; Figure 1, Supporting Information S1: Material 5) and better alliances with the therapists (*r*_*b*_ = 0.60‐−62; Figure 1, Supporting Information S1: Material 6). However, the relationship between patient IB (as judged by therapists) and the patients' perspective on the alliance was not as consistent as when considering only the therapists' perspectives (Figure 1, Supporting Information S1: Material 7). e.g., although the patients' judgments of the alliance were significantly correlated with the therapists' judgments of patient IB in most sessions (i.e., 11 of 20 sessions), there were also several sessions without significant effects (e.g., nine of 20 sessions in the session‐to‐session interval and four of five sessions in the four‐session interval). However, the between‐person level exhibited a positive correlation (*r*_*b*_ = 0.34–38), indicating that patients who generally reported better alliances were also perceived as demonstrating more positive behaviors by their therapists (Figure 1, Supporting Information S1: Material 7).

Positive Cross‐Lagged Effects from Session to Session.

Overall, H2 was only partly confirmed because the results differed depending on the perspective and time interval. From the perspective of the raters, H2 could not be confirmed, as shown by the non‐significant correlations between the deviations in the patient's IB in one session and the therapist's competence four sessions later (or vice versa; Figure 1, Supporting Information S1: Material 5).

However, H2 was partly confirmed from the perspective of the therapists. As Figure 1 shows, there were significant (constrained) cross‐lagged effects in the session‐to‐session interval but not in the four‐session interval (Supporting Information S1: Material 6). This indicated that deviations from therapists' perceived alliance were associated with deviations in patient IB one session before (*B* = 0.11). This effect was reciprocal, meaning that deviations in alliances were also positively associated with how the therapists perceived their patients' IB one session later (*B* = 0.08).

Considering the patient perspective (Figure 1, Supporting Information S1: Material 7), there were some significant cross‐lagged effects in the session‐to‐session interval, which generally corresponded with the therapist‐only perspective. However, there was either no clear pattern (i.e., with both negative and positive correlations between therapist rated patient IB and patient rated alliance) or only sporadic effects (i.e., positive cross‐lagged effects for only five sessions). As with the therapists, there were no significant cross‐lagged effects across the four‐session intervals. Therefore, we cannot confirm H2, as the patients' views on the alliance in a session were not significantly associated with therapist rated patient IB, neither one session nor four sessions later.

Negative Correlations with Interpersonal Problems Before Therapy.

H3 could not be confirmed from any perspective. As Figure 1 and Supporting Information S1: Materials 5 to 7 show, patients with a higher level of interpersonal problems before therapy did not report having therapists with lower competences (rater perspective) or worse therapeutic alliances (therapist or patient perspectives). Unexpectedly, raters found that patients with higher levels of interpersonal problems before treatment showed significantly more positive IB (*B* = 0.17; Figure 1, Supporting Information S1: Materials 5).

### 3.2 Additional Results

The control variable competence feedback (vs. no feedback) was significantly correlated with the rater‐based competence scores (*B* = 0.45) but not the patient‐ or therapist‐based therapeutic alliance scores (therapist and patient–therapist models; Figure 1, Supporting Information S1: Materials 5 to 7).

We also included the level of patients' interpersonal problems after treatment as an outcome variable in the RI‐CLPMs (Supporting Information S1: Materials 5 to 7). As Figure 1 shows, none of rater‐based variables (patient IB, therapist competence) was significantly associated with interpersonal problems after treatment (Supporting Information S1: Material 6). However, the therapists' positive evaluations of their patients' IB were significantly associated with lower levels of interpersonal problems after therapy (in both time intervals, *Bs* = −0.24 to −0.29; Supporting Information S1: Material 8). Yet, the therapists' evaluation of the alliance was not significantly associated with the level of interpersonal problems after therapy (Figure 1). Patients' evaluations of the alliance were not significantly associated with their general level of interpersonal problems after therapy (Figure 1, Supporting Information S1: Material 7). However, the level of interpersonal problems before treatment was positively associated with the level of interpersonal problems after treatment across all models (*Bs* = 0.54 to 0.60; Figure 1).

Figure 1 presents the results of the autoregressive effects, which were not part of our main hypotheses. The raters perceived stable deviations only in patients' IB (*B*s = 0.21 to 0.31), not in therapist competence, across all four measurement points. However, therapists found deviations in their patients' IB to be stable across some subsequent sessions (*B*s = 0.21–0.50 for sessions 1–5, sessions 8–10, and sessions 14–20) but not across larger four‐session intervals. Similarly, the therapists found deviations in the therapeutic alliance to be stable in most subsequent sessions (*B*s = 0.21–0.53 for sessions 1–4 and sessions 8–20) but only in two of four larger four‐session intervals (*B*s = 0.43–0.51 for intervals 9–13 and intervals 13–17). Like the therapists did, the patients perceived deviations in the therapeutic alliance to be stable primarily in the beginning and end phases of therapy (*B*s = 0.26–0.69 for sessions 1–9 and sessions 13–20). However, unlike for the therapists, this stability did not hold over longer four‐session intervals since there was only one significant autoregressive effect (*B* = 0.24 in intervals 1–5).

## 4 Discussion

The aim of the present study was to better understand the interaction between therapist and patient during therapy. Using a longitudinal design, we were able to relate patient IB to therapist competence and the alliance over 24 sessions of CBT. To obtain a differentiated picture, we looked at the relationships from a micro‐ and a mesoperspective (each session vs. every 4th session), and from three different assessment perspectives (i.e., rater, therapist, and patient). We also considered the influence of the general level of patient interpersonal problems before therapy. In the following, we discuss the results of our three hypotheses.

### 4.1 Within‐ and Between‐Person Correlations Between the Process Variables

Our main hypothesis was that positive patient IB significantly correlates with better therapist competence and therapeutic alliances (H1). The positive intercorrelations in this study indicated that intraindividual deviations in patient IB in one session were associated with intraindividual deviations in therapist competence or alliance in the same session. In addition, patients with more positive IBs generally experienced not only better alliances but also more competent therapists (between‐person). Consequently, H1 was confirmed both intra‐ and interindividually. This finding is in line with previous findings on therapist‒patient interactions showing that the three variables are mutually dependent (Foley et al. 1987; Gazzillo et al. 2018; Henry et al. 1986; Huber et al. 2019, 2021; McFarquhar et al. 2018; Richtberg et al. 2016; Westra et al. 2012; Zickgraf et al. 2016). Furthermore, the present study confirmed that patient IB is an important component of therapist competence and the alliance, that therapist and patient behavior are mutually dependent, and that all behavior invites a reaction (McFarquhar et al. 2018).

However, it is important to note that the within‐person correlations were less consistent in the model that combined therapist and patient perspectives. This result might indicate a potential overlap of criteria at the construct level (cf. results of Gazzillo et al. 2018). Raters and therapists might perceive similar anchors for patient IB and therapist competence or the alliance, especially when evaluating both constructs simultaneously, increasing the risk of perception bias. Both raters and therapists might, therefore, automatically attribute deficits in alliance or competence to dysfunctional patient IB—just as they might automatically attribute positive patient IB to increased therapist competence or alliance. Future studies should explore the possibility of conceptual overlap and potential perception biases further by comparing assessments of patient IB with those of other individuals who did not simultaneously assess competence or the alliance. In addition, new measurements might be needed that clearly delineate specific criteria and that accommodate potential perception biases to refine the assessment process in therapeutic settings.

### 4.2 Cross‐Lagged Effects and the Role of Perspective and Time Frame

Our second hypothesis was that positive patient IB in one session is correlated with improved therapist competence and therapeutic alliances in subsequent sessions and vice versa (H2). By analyzing cross‐lagged effects, we investigated the extent to which intra‐individual changes, for example on the patient side, can be predicted by previous intra‐individual changes on the therapist side. This hypothesis was only partially confirmed, specifically concerning therapists' evaluations of patient IB and the therapeutic alliance, and only in the session‐to‐session interval. This should be interpreted together with the fact that there were inconsistent cross‐lagged effects when considering the patients' perspective on the therapeutic alliance (i.e., in the patient‒therapist models). This discrepancy suggests again that therapists may be prone to perception bias. Therapists might interpret negative patient IBs as a sign of a bad alliance and positive IBs as indicative of a strong alliance, even though patients themselves do not report worse or better alliance when exhibiting this behavior. Similarly, therapists might interpret negative alliances as signs of negative behaviors and good alliances as indicators of positive patient IBs. It is well known that therapists use several intuitive and fast‐thinking processes, such as confirmation bias or other heuristics (Tversky and Kahneman 1974). It is possible that such biases could occur without consequences for actual behavior. However, therapist biases can also “contribute to illogical thinking, affect medical decision making, and adversely affect the conduct of psychotherapy” (Yager et al. 2021, p. 119). Importantly, the sample consisted of trainees, some of whom were treating their first outpatients. Accordingly, the results relate to relatively inexperienced therapists who need specific support in interpreting the dynamics of therapy appropriately and not allowing themselves to be overly influenced by one of many explanations for negative patient IB. However, the impact seems to fade over time, as indicated by the nonsignificant cross‐lagged effects at the four‐session interval. Therefore, the differentiation of smaller and larger time intervals seems promising for expanding the knowledge about the relationships among variables in the therapy process (Coyne et al. 2019; Flückiger et al. 2020). In addition, future studies should include additional independent observers to assess the alliance from one session to the next to investigate whether this bias also translates into objectively observable behavior. Nevertheless, training programs could be developed to help therapists recognize and avoid unintentional biases. These programs may focus on enhancing the accuracy and objectivity of individual patient assessments.

However, H2 was rejected in all other models (the interrater perspective, therapist perspective during the four‐session interval, and patient‐therapist perspective during both time intervals). One explanation for the (nonsignificant) results from the rater perspective might be that even sessions characterized by lower therapist competence are not automatically associated with negative patient behavior in the long term. Conversely, therapist competence seems to remain unaffected by the patients' previous IB. This idea is supported by a previous study that did not find any significant relationships between the AFPIB and the CTS across a time interval of six sessions (Weck et al. 2016). The lack of such relationships may suggest that they are quite modest in size, assuming that the methods and powers of both studies were appropriate for capturing the effects of interest. However, another explanation could be that the four‐session interval was too large to detect relationships. Future research should investigate the stability and situational variations in therapist competence at different time intervals (Weck 2013).

In summary, this study demonstrated the crucial nature of the questions of who is evaluating the interaction between therapist and patient and at which time interval. The differences between the models may indicate a potential risk of bias when therapists and raters assess both variables simultaneously (i.e., patient IB and competence or patient IB and alliance). For this reason, therapists should be aware of potential perception biases when evaluating such variables simultaneously, and doing so may encourage researchers and practitioners to adopt multimethod assessments. Additionally, assessment tools that address these biases and incorporate diverse perspectives may need to be revised or developed.

### 4.3 The Role of Patients' Interpersonal Problems

Finally, we hypothesized that patients with greater interpersonal problems before therapy would exhibit more negative patient IB, encounter therapists with lower competencies, and experience weaker alliances (H3). Unexpectedly, the process variables were largely independent of the patients' level of interpersonal problems before treatment—with the exception of the rater's perspective. On the one hand, these results are encouraging, as patients with severe interpersonal problems can expect similarly competent therapists and similarly good alliances as patients with fewer initial problems. From the raters' perspective, these patients even showed more positive IB in the therapeutic sessions (instead of negative, as assumed). It therefore also seems plausible that such patients put a lot of effort into therapy and thus benefit more from it in the long term. However, the effects were small and should therefore be interpreted with caution. Another explanation for the results in this study could be that the sample had rather low scores on the IIP and also showed more positive scores on the AFPIB, which may have distorted the relationships somewhat. In addition, the positive correlations of interpersonal problems before and after treatment indicate a fundamentally relative stability of the construct, which at least does not seem to change too much as a result of the CBT treatment presented here. Finally, we only considered the total score of the IIP. However, there is evidence that the subscales show differential effects (i.e., agency vs. communion; Constantino et al. 2016; Puschner et al. 2004). Future studies might therefore explore the relationships in more disturbed samples and differentiate between IIP subscales.

### 4.4 The Role of Competence Feedback and the Stability of Autoregressive Effects

As the present study was a secondary analysis of an RCT, we included the group condition as a control variable. The study group (competence feedback vs. no feedback) revealed a significant effect in favor of competence feedback only for the rater‐based CTS scores, which is in line with previous research (Weck et al. 2017, 2021). The significant autoregressions in this study indicated stability of intraindividual changes only for patient IB and the alliance (but not rater‐based competence scores). In addition, the results also suggest that such intraindividual variations are mostly stable in the beginning and end phases of the therapy. However, this also means that intraindividual deterioration in patients' IB and the alliance are relatively stable during these phases and may be more difficult to change. Therefore, targeted interventions, such as motivational clarification or social skills training, may be needed to directly address a change.

### 4.5 Limitations and Future Research

Although the present study has numerous strengths (e.g., multiple perspectives, session‐to‐session analyses, and a sophisticated statistical model), several limitations need to be considered when conclusions are drawn. First, the AFPIB is an observer‐based instrument; hence, a comparison between self‐ and observer‐assessed patient IBs in future studies is desirable. Future studies should also compare the results with more complex measurements used in the frameworks of the Interpersonal Circumplex Model (Horowitz et al. 2000) or the Structural Analysis of Social Behavior Models (Henry et al. 1986). Second, we did not examine to what extent the intrapersonal variations in the investigated variables were also associated with treatment outcomes (e.g., variations in depressive symptoms). Third, as this is a secondary analysis, we did not plan the sample size with an appropriate a priori power analysis. However, we followed recommendations in the literature (Falkenström et al. 2022; Zyphur et al. 2020) to increase statistical power. Nonetheless, the results require replication in adequately powered study designs. Fourth, the comparability of the results between raters and therapists is limited because the analyses for the session‐to‐session interval and for the alliance were missing. However, because the original study was not designed for the present research question, these data were not available to us. In this context, it is also important to recognize that most findings relied on therapists' assessments. Future research should aim to balance the perspectives of raters, therapists, and patients. Fifth, future studies could replicate the findings in samples of patients with mood disorders other than major depression, such as persistent depressive disorder. Finally, the therapists in this study were still in training, which could have influenced their ability to evaluate patients′ IB accurately or establish strong therapeutic alliances. Consequently, future studies should replicate the findings in samples with experienced therapists.

### 4.6 Practical Implications

Despite these limitations, this study has important implications for clinical practice and researchers in the field of therapist competence. Given that therapists in particular felt that their current alliance was associated with their patients′ IB in previous sessions, therapists should be aware that this could be a misinterpretation along the following lines: “If the behavior is problematic/positive, then our relationship is bad/good.” Consequently, therapists should engage in supervision, mindfulness, and self‐reflection to better assess the current ongoing dynamics in the therapy room (e.g., Henry et al. 1986). Additionally, education on therapist biases could improve the accuracy and validity of assessments regarding patient IB and the therapeutic alliance. Offering appropriate information before evaluations may help mitigate these biases. In addition, it seems important that therapists monitor the alliance, especially from the patient′s perspective. Given the connection between patient IB and therapeutic competences, researchers might consider patients' IB more often when assessing competence to account for the overlap. Future studies should also investigate the question of potential perception bias when simultaneously evaluating patient IB and the alliance.

## 5 Conclusions

The present study supports the idea that patient IB is an important part of both therapeutic competence and alliance (and vice versa). Patients with generally more positive IB experience both more competent therapists and better relationships. Furthermore, intraindividual changes in the level of IB are also associated with changes in competence and alliance. Moreover, intraindividual changes in patient IB, at least from the therapist's perspective, also predict intraindividual changes in alliance from session to session, just as an intraindividual change in perceived alliance is associated with a change in the perception of patient IB in the following session. It is important to keep in mind, however, that if the same person assesses patient and therapist behavior simultaneously, he or she may be prone to assessment biases. In this case, therapists may tend to automatically (and possibly incorrectly) interpret negative patient IB as a sign of poor alliance (and vice versa). Finally, the level of interpersonal problems before treatment does not appear to significantly influence the three process variables—suggesting that patients with both more and fewer social problems may have similar experiences in therapy.

## Ethics Statement

Ethical approval was obtained from the Institutional Review Board of the University of Potsdam (No. 31/2016).

## Consent

All the participants provided written informed consent.

## Conflicts of Interest

The authors declare no conflicts of interest.

## Supporting information


**Supplementary Material 1:** Example of the Random‐Intercept Cross‐Lagged Panel Model with Time‐Invariant Constraints and Five Measurement Points. **Supplementary Material 2:** Model Comparisons for the Rater Perspective (Four‐Session Interval). **Supplementary Material 3:** Model Comparisons for the Therapist Perspective. **Supplementary Material 4:** Model Comparisons for the Patient‐Therapist Perspective. **Supplementary Material 5:** Rater Perspective: Results of the RI‐CLPM for the Interplay of Patient Interpersonal Behavior and Therapeutic Competence (Four‐Session Interval). **Supplementary Material 6:** Therapist Perspective: Results of the RI‐CLPM for the Interplay Between Patient Interpersonal Behavior and the Therapeutic Alliance. **Supplementary Material 7:** Considering the Patient Perspective: Results of the RI‐CLPM for the Interplay Between Patient Interpersonal Behavior (Therapists’ Perspective) and the Therapeutic Alliance (Patients’ Perspective).

## Data Availability

The data are not publicly available due to privacy or ethical restrictions. The data that support the findings of this study are available from the corresponding author upon reasonable request. The analysis codes are available at the Open Science Framework: https://doi.org/10.17605/OSF.IO/69CMQ.

## Endnotes

1 The choice of model did not affect the interpretation of the results.
