# Supplementary material for: Relationships Among Patients' Interpersonal Behaviors in Sessions, Therapist Competence, and the Therapeutic Alliance in Cognitive Behavior Therapy: A Cross‐Lagged Panel Analysis
Source: J Clin Psychol. 2025 Aug 30;82(1):18–30. doi: 10.1002/jclp.70040 (PMC12688284; doi:10.1002/jclp.70040)
Supplement: Supplementary file 1 — Supplementary Material 1: Example of the Random‐Intercept Cross‐Lagged Panel Model with Time‐Invariant Constraints and Five Measurement Points. Supplementary Material 2: Model Comparisons for the Rater Perspective (Four‐Session Interval). Supplementary Material 3: Model Comparisons for the Therapist Perspective. Supplementary Material 4: Model Comparisons for the Patient‐Therapist Perspective. Supplementary Material 5: Rater Perspective: Results of the RI‐CLPM for the Interplay of Patient Interpersonal Behavior and Therapeutic Competence (Four‐Session Interval). Supplementary Material 6: Therapist Perspective: Results of the RI‐CLPM for the Interplay Between Patient Interpersonal Behavior and the Therapeutic Alliance. Supplementary Material 7: Considering the Patient Perspective: Results of the RI‐CLPM for the Interplay Between Patient Interpersonal Behavior (Therapists’ Perspective) and the Therapeutic Alliance (Patients’ Perspective). [file JCLP-82-18-s001.docx]

## Supplementary Material 1

*Example of the Random-Intercept Cross-Lagged Panel Model with Time-Invariant Constraints and Five Measurement Points*

*
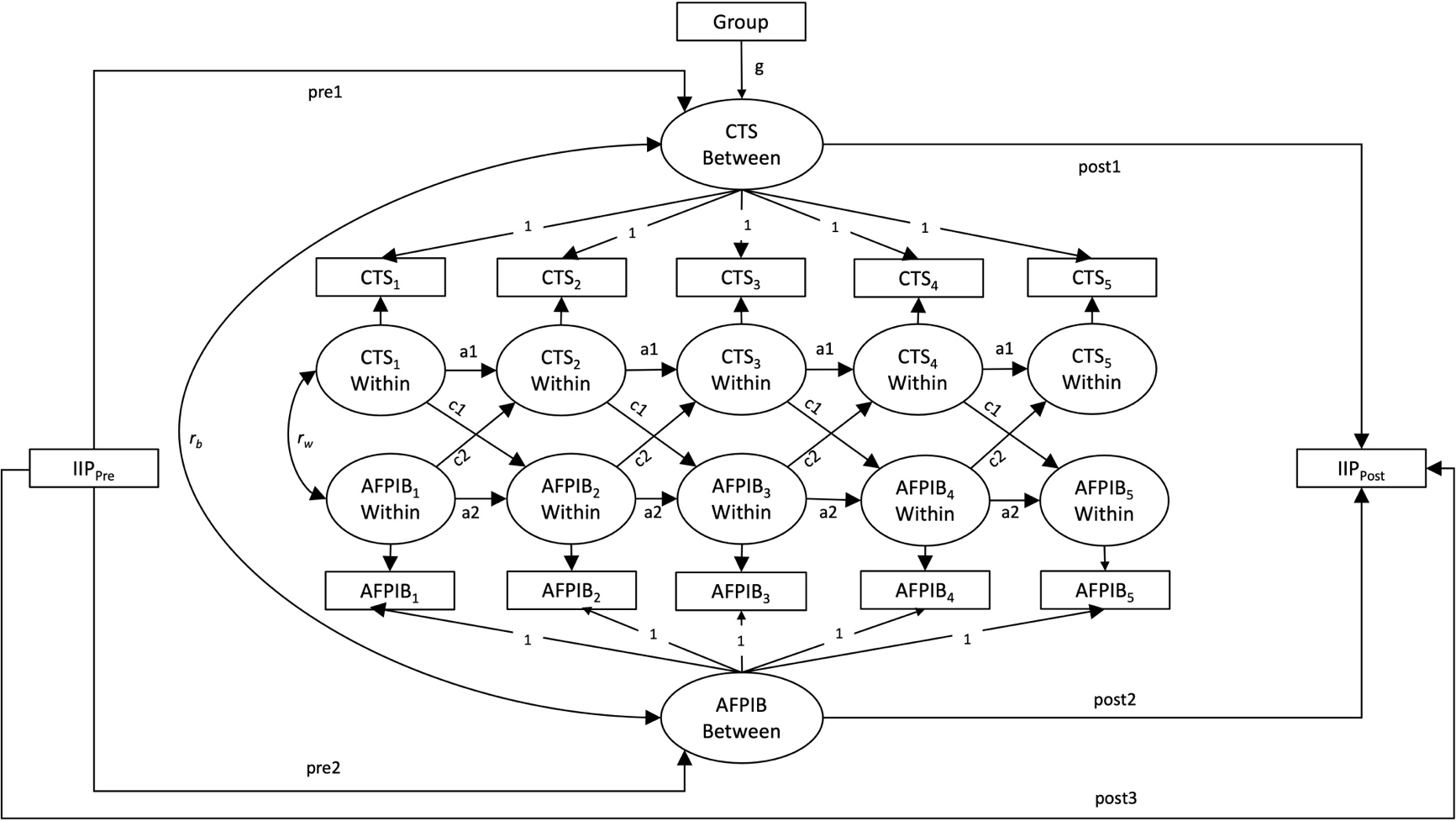
*

*Note.* Group = study group (feedback vs. no feedback). CTS = Cognitive Therapy Scale (therapeutic competence). Between = between-person level. Within = within-person level. AFPIB = Assessment of Patient Interpersonal Behavior Form (patient interpersonal behavior). IIP_pre_ / IIP_post_ = Scores of the Inventory of Interpersonal Problems, pretreatment and posttreatment.

## Supplementary Material 2

*Model Comparisons for the Rater Perspective (Four-Session Interval)*

|  | *df* | *AIC* | *Χ^2^* | *p* |
| --- | --- | --- | --- | --- |
| Basic Model | 57 | 1324.5 | 74.17 |  |
| *Test for Constraints* |  |  |  |  |
| + constrained autoregressions | 53 | 1317.5 | 79.16 | .329 |
| + constrained cross-lagged effects | **59** | **1313.0** | **86.61** | **.478** |
| + constrained (co-)variances | 68 | 1316.1 | 107.71 | .012 |
| *Test for Time Trends* |  |  |  |  |
| Non-Detrended | **67** | **1303.7** | **93.26** | **.575** |
| *Compare with Observational Approach* |  |  |  |  |
| Observational Approach | **63** | **1297.9** | **79.53** |  |
| Residual Approach | 67 | 1303.7 | 93.26 | .008 |

## Supplementary Material 3

*Model Comparisons for the Therapist Perspective*

|  | **Session-to-Session Interval** | | | |  | **Four-Session Interval** | | | |
| --- | --- | --- | --- | --- | --- | --- | --- | --- | --- |
|  | *df* | *AIC* | *Χ^2^* | *p* |  | *df* | *AIC* | *Χ^2^* | *p* |
| Basic Model | 797 | 3338.1 | 1218.0 |  |  | 47 | 1312.1 | 71.48 |  |
| *Test for Constraints* | | | | | | | | | |
| + constrained autoregressions | 833 | 3318.1 | 1270 | .041 |  | 53 | 1314.0 | 85.39 | .031 |
| + constrained cross-lagged effects | **833** | **3312.3** | **1264.3** | **.118** |  | **53** | **1304.8** | **76.19** | **.582** |
| *Test for Time Trends* | | | | | | | | | |
| Non-Detrended | 871 | 3346.1 | 1374.0 | <.001 |  | 61 | 1331.8 | 119.13 | <.001 |
| *Compare with Observational Approach* | | | | | | | | | |
| Observational Approach | **829** | **3289.8** | **1233.7** |  |  | **49** | **1295.9** | **59.20** |  |
| Residual Approach | 833 | 3312.3 | 1264.3 | <.001 |  | 53 | 1304.8 | 76.19 | .002 |

## Supplementary Material 4

*Model Comparisons for the Patient-Therapist Perspective*

|  | **Session-to-Session Interval** | | | |  | **Four-Session Interval** | | | |
| --- | --- | --- | --- | --- | --- | --- | --- | --- | --- |
|  | *df* | *AIC* | *Χ^2^* | *p* |  | *df* | *AIC* | *Χ^2^* | *p* |
| Basic Model | **797** | **3617.3** | **1451.0** |  |  | 47 | 1414.3 | 98.23 |  |
| *Test for Constraints* |  |  |  |  |  |  |  |  |  |
| + constrained autoregressions | 833 | 3637.3 | 1543.1 | <.001 |  | 53 | 1414.4 | 110.33 | .060 |
| + constrained cross-lagged | 833 | 3607.2 | 1513 | <.001 |  | **53** | **1407.3** | **103.25** | **.541** |
| *Test for Time Trends* |  |  |  |  |  |  |  |  |  |
| Non-Detrended | 835 | 3644.8 | 1554.6 | <.011 |  | 61 | 1434.6 | 146.53 | <.001 |
| *Compare with Observational Approach* |  |  |  |  |  |  |  |  |  |
| Observational Approach | **793** | **3582.5** | **1408.2** |  |  | **49** | **1393.2** | **81.12** |  |
| Residual Approach | 797 | 3617.3 | 1451.0 | <.001 |  | 53 | 1407.3 | 103.25 | <.001 |

## Supplementary Material 5

*Rater Perspective: Results of the RI-CLPM for the Interplay of Patient Interpersonal Behavior and Therapeutic Competence (Four-Session Interval)*

|  | ***β (SE)*** | ***B*** | ***p*** |
| --- | --- | --- | --- |
| *Within-Person Level* |  |  |  |
| pib-pib | **0.24 (0.08)** | **.21**–**.31** | **.002** |
| cts-cts | 0.06 (0.07) | .05–.07 | .406 |
| pib_t_ -> cts_t+1_ | -0.05 (0.10) | -.03– -.04 | .635 |
| cts_t_ -> pib_t+1_ | 0.02 (0.05) | .02– .03 | .708 |
| r_w_ |  |  |  |
| 1 | **0.04 (0.02)** | **.25** | **.041** |
| 2 | **0.04 (0.02)** | **.35** | **.008** |
| 3 | **0.04 (0.02)** | **.29** | **.026** |
| 4 | **0.06 (0.02)** | **.49** | **.001** |
| 5 | **0.04 (0.02)** | **.37** | **.008** |
| *Between-Person Level* |  |  |  |
| ip_pre_ -> pib | **0.14 (0.07)** | **.17** | **.047** |
| ip_pre_ -> cts | 0.08 (0.09) | .09 | .333 |
| pib -> ip_post_ | -0.13 (0.14) | -.10 | .357 |
| cts -> ip_post_ | -0.03 (0.13) | .03 | .807 |
| iip_pre_ -> ip_post_ | **0.69 (0.10)** | **.60** | **<.001** |
| group -> cts | **0.41 (0.09)** | **.45** | **<.001** |
| r_b_ | **0.08 (0.02)** | **.47** | **<.001** |

*Note*. Auto-regressive and cross-lagged effects are constrained. cts = cognitive therapy scale (therapeutic competence). pib = patient interpersonal behavior. Group = study group (competence vs. no feedback). r_b_ = between-person correlation. t = a session at a given time point. t+1 = subsequent session. r_w_ = correlation within sessions.

## Supplementary Material 6

*Therapist Perspective: Results of the RI-CLPM for the Interplay Between Patient Interpersonal Behavior and the Therapeutic Alliance*

|  | **Session-to-Session Interval** | | | **Four-Session Interval** | | |
| --- | --- | --- | --- | --- | --- | --- |
|  | *β (SE)* | *B* | *p* | *β (SE)* | *B* | *p* |
| *Within-Person Level* | | | | | | |
| pib-pib |  |  |  |  |  |  |
| 1-2 | **0.51 (0.08)** | **.50** | **<.001** | 0.07 (0.11) | .08 | .550 |
| 2-3 | **0.47 (0.07)** | **.48** | **<.001** | -0.23 (0.18) | -.22 | .214 |
| 3-4 | **0.23 (0.08)** | **.26** | **.003** | 0.05 (0.17) | .06 | .746 |
| 4-5 | **0.20 (0.08)** | **.22** | **.013** | 0.24 (0.15) | .20 | .109 |
| 5-6 | 0.003 (0.11) | .003 | .976 |  |  |  |
| 6-7 | 0.12 (0.09) | .13 | .148 |  |  |  |
| 7-8 | 0.16 (0.10) | .15 | .110 |  |  |  |
| 8-9 | **0.20 (0.09)** | **.22** | **.021** |  |  |  |
| 9-10 | **0.20 (0.09)** | **.21** | **.018** |  |  |  |
| 10-11 | 0.14 (0.10) | .14 | .141 |  |  |  |
| 11-12 | 0.17 (0.09) | .19 | .068 |  |  |  |
| 12-13 | 0.20 (0.10) | .19 | .052 |  |  |  |
| 13-14 | 0.15 (0.12) | .13 | .188 |  |  |  |
| 14-15 | **0.26 (0.09)** | **.26** | **.006** |  |  |  |
| 15-16 | **0.24 (0.10)** | **.21** | **.023** |  |  |  |
| 16-17 | **0.28 (0.08)** | **.40** | **<.001** |  |  |  |
| 17-18 | **0.44 (0.08)** | **.45** | **<.001** |  |  |  |
| 18-19 | **0.30 (0.09)** | **.30** | **.001** |  |  |  |
| 19-20 | **0.43 (0.11)** | **.42** | **<.001** |  |  |  |
| haq-haq |  |  |  |  |  |  |
| 1-2 | **0.33 (0.08)** | **.26** | **<.001** | 0.15 (0.17) | .13 | .371 |
| 2-3 | **0.34 (0.10)** | **.32** | **<.001** | 0.28 (0.18) | .28 | .111 |
| 3-4 | **0.33 (0.08)** | **.35** | **<.001** | **0.55 (0.13)** | **.51** | **.000** |
| 4-5 | 0.19 (0.10) | .18 | .056 | **0.45 (0.13)** | **.43** | **.001** |
| 5-6 | 0.09 (0.10) | .09 | .365 |  |  |  |
| 6-7 | 0.13 (0.08) | .15 | .106 |  |  |  |
| 7-8 | 0.09 (0.10) | .09 | .366 |  |  |  |
| 8-9 | **0.31 (0.10)** | **.31** | **.002** |  |  |  |
| 9-10 | **0.23 (0.10)** | **.21** | **.019** |  |  |  |
| 10-11 | **0.42 (0.09)** | **.41** | **<.001** |  |  |  |
| 11-12 | **0.29 (0.07)** | **.39** | **<.001** |  |  |  |
| 12-13 | **0.34 (0.12)** | **.28** | **.004** |  |  |  |
| 13-14 | **0.55 (0.10)** | **.47** | **<.001** |  |  |  |
| 14-15 | **0.43 (0.07)** | **.49** | **<.001** |  |  |  |
| 15-16 | **0.37 (0.11)** | **.32** | **<.001** |  |  |  |
| 16-17 | **0.32 (0.09)** | **.33** | **<.001** |  |  |  |
| 17-18 | **0.45 (0.10)** | **.40** | **<.001** |  |  |  |
| 18-19 | **0.50 (0.08)** | **.51** | **<.001** |  |  |  |
| 19-20 | **0.40 (0.08)** | **.53** | **<.001** |  |  |  |
| pib_t_->haq_t+1_ | **0.09 (0.03)** | **.11** | **.001** | -0.05 (0.09) | -.04 | .590 |
| haq_t_->pib_t+1_ | **0.08 (0.03)** | **.08** | **.005** | 0.16 (0.09) | .16–.19 | .079 |
| r_w_ |  |  |  |  |  |  |
| 1 | 0.07 (0.02) | **.42** | **<.001** | 0.04 (0.03) | .28 | .128 |
| 2 | 0.06 (0.01) | **.44** | **<.001** | **0.10 (0.02)** | **.70** | **.002** |
| 3 | 0.06 (0.02) | **.48** | **<.001** | **0.09 (0.03)** | **.57** | **<.001** |
| 4 | 0.08 (0.01) | **.63** | **<.001** | **0.09 (0.02)** | **.63** | **<.001** |
| 5 | 0.08 (0.02) | **.65** | **<.001** | **0.11 (0.02)** | **.62** | **<.001** |
| 6 | 0.08 (0.02) | **.55** | **<.001** |  |  |  |
| 7 | 0.08 (0.02) | **.64** | **<.001** |  |  |  |
| 8 | 0.08 (0.02) | **.62** | **<.001** |  |  |  |
| 9 | 0.06 (0.01) | **.50** | **<.001** |  |  |  |
| 10 | 0.08 (0.02) | **.65** | **<.001** |  |  |  |
| 11 | 0.07 (0.01) | **.62** | **<.001** |  |  |  |
| 12 | 0.03 (0.01) | **.38** | **.001** |  |  |  |
| 13 | 0.06 (0.01) | **.60** | **<.001** |  |  |  |
| 14 | 0.09 (0.02) | **.67** | **<.001** |  |  |  |
| 15 | 0.07 (0.01) | **.62** | **<.001** |  |  |  |
| 16 | 0.10 (0.02) | **.61** | **<.001** |  |  |  |
| 17 | 0.08 (0.02) | **.60** | **<.001** |  |  |  |
| 18 | 0.09 (0.02) | **.64** | **<.001** |  |  |  |
| 19 | 0.08 (0.02) | **.59** | **<.001** |  |  |  |
| 20 | 0.05 (0.01) | **.40** | **.001** |  |  |  |
| *Between-Person Level* | | | | | | |
| ip_pre_ -> pib | -0.01 (0.08) | -.01 | .872 | -0.04 (0.08) | -.04 | .647 |
| ip_pre_ -> haq | -0.07 (0.08) | -.09 | .337 | -0.09 (0.08) | -.14 | .248 |
| pib -> ip_post_ | **-0.33 (0.12)** | **-.29** | **.008** | **-0.28 (0.14)** | **-.24** | **.040** |
| haq -> ip_post_ | 0.12 (0.16) | .08 | .470 | -0.07 (0.25) | -.04 | .780 |
| iip_pre_ -> ip_post_ | **0.67 (0.09)** | **.58** | **<.001** | **0.65 (0.09)** | **.57** | **<.001** |
| group -> haq | 0.10 (0.06) | .12 | .115 | 0.09 (0.06) | .14 | .161 |
| r_b_ | **0.12 (0.03)** | **.62** | **<.001** | **0.09 (0.03)** | **.60** | **.008** |

*Note*. Auto-regressive and cross-lagged effects are constrained. haq = helping alliance questionnaire (therapeutic alliance). PIB = patient interpersonal behavior. Group = study group (competence vs. no feedback). r_b_ = between-person correlation. t = a session at a given time point. t+1 = subsequent session. r_w_ = correlation within sessions.

## Supplementary Material 7

*Considering the Patient Perspective: Results of the RI-CLPM for the Interplay Between Patient Interpersonal Behavior (Therapists’ Perspective) and the Therapeutic Alliance (Patients’ Perspective)*

|  | **Session-to-Session Interval** | | | **Four-Session Interval** | | |
| --- | --- | --- | --- | --- | --- | --- |
|  | *β (SE)* | *B* | *p* | *β (SE)* | *B* | *p* |
| *Within-Person Level* | | | | | | |
| pib-pib |  |  |  |  |  |  |
| 1-2 | **0.57 (0.09)** | **.54** | **<.001** | 0.04 (0.15) | .05 | .797 |
| 2-3 | **0.44 (0.08)** | **.47** | **<.001** | -0.15 (0.22) | -.14 | .516 |
| 3-4 | **0.31 (0.10)** | **.33** | **.001** | 0.09 (0.19) | .09 | .627 |
| 4-5 | **0.28 (0.10)** | **.31** | **.003** | **0.46 (0.16)** | **.36** | **.003** |
| 5-6 | -0.04 (0.12) | -.04 | .707 |  |  |  |
| 6-7 | 0.06 (0.11) | .06 | .600 |  |  |  |
| 7-8 | **0.27 (0.09)** | **.23** | **.043** |  |  |  |
| 8-9 | **0.27 (0.09)** | **.29** | **.004** |  |  |  |
| 9-10 | **0.35 (0.11)** | **.37** | **.001** |  |  |  |
| 10-11 | **0.27 (0.11)** | **.27** | **.011** |  |  |  |
| 11-12 | **0.19 (0.09)** | **.23** | **.037** |  |  |  |
| 12-13 | 0.10 (0.13) | .10 | .418 |  |  |  |
| 13-14 | 0.22 (0.14) | .18 | .117 |  |  |  |
| 14-15 | 0.16 (0.11) | .17 | .138 |  |  |  |
| 15-16 | 0.25 (0.15) | .22 | .083 |  |  |  |
| 16-17 | **0.34 (0.10)** | **0.36** | **<.001** |  |  |  |
| 17-18 | **0.46 (0.09)** | **0.46** | **<.001** |  |  |  |
| 18-19 | **0.40 (0.12)** | **0.43** | **.001** |  |  |  |
| 19-20 | **0.37 (0.13)** | **0.35** | **.004** |  |  |  |
| haq-haq |  |  |  |  |  |  |
| 1-2 | **0.43 (0.07)** | **.54** | **<.001** | **0.21 (0.10)** | **.24** | **.043** |
| 2-3 | **0.41 (0.10)** | **.39** | **<.011** | -0.13 (0.12) | -.19 | .300 |
| 3-4 | **0.36 (0.09)** | **.39** | **<.001** | -0.43 (0.30) | -.42 | .153 |
| 4-5 | **0.34 (0.10)** | **.34** | **.001** | -0.02 (0.28) | -.01 | .947 |
| 5-6 | **0.23 (0.11)** | **.27** | **.031** |  |  |  |
| 6-7 | **0.51 (0.13)** | **.39** | **<.001** |  |  |  |
| 7-8 | **0.40 (0.10)** | **.43** | **<.001** |  |  |  |
| 8-9 | **0.20 (0.08)** | **.26** | **.012** |  |  |  |
| 9-10 | 0.05 (0.14) | .04 | .743 |  |  |  |
| 10-11 | -0.01 (0.09) | -.02 | .890 |  |  |  |
| 11-12 | -.004 (0.12) | -.004 | .975 |  |  |  |
| 12-13 | 0.08 (0.12) | .08 | .493 |  |  |  |
| 13-14 | **0.38 (0.14)** | **.32** | **.005** |  |  |  |
| 14-15 | **0.42 (0.10)** | **.43** | **<.001** |  |  |  |
| 15-16 | **0.33 (0.15)** | **.28** | **.025** |  |  |  |
| 16-17 | **0.54 (0.11)** | **.28** | **<.001** |  |  |  |
| 17-18 | **0.73 (0.08)** | **.69** | **<.001** |  |  |  |
| 18-19 | **0.21 (0.10)** | **.27** | **.036** |  |  |  |
| 19-20 | **0.57 (0.12)** | **.56** | **<.001** |  |  |  |
| pib_t_->haq_t+1_ |  |  |  |  |  |  |
| 1-2 | 0.02 (0.07) | .03 | .778 | 0.01 (0.06) | .01 | .851 |
| 2-3 | -0.11 (0.08) | -.14 | .153 | 0.01 (0.06) | .02 | .851 |
| 3-4 | 0.04 (0.08) | .05 | .639 | 0.01 (0.06) | .02 | .851 |
| 4-5 | -0.14 (0.08) | -.17 | .099 | 0.01 (0.06) | .01 | .851 |
| 5-6 | **-0.23 (0.10)** | **-.25** | **.014** |  |  |  |
| 6-7 | -0.02 (0.12) | -.02 | .879 |  |  |  |
| 7-8 | -0.19 (0.12) | -.17 | .118 |  |  |  |
| 8-9 | -0.11 (0.08) | -.15 | .158 |  |  |  |
| 9-10 | 0.01 (0.09) | .01 | .957 |  |  |  |
| 10-11 | 0.07 (0.09) | .09 | .435 |  |  |  |
| 11-12 | 0.05 (0.09) | .07 | .566 |  |  |  |
| 12-13 | 0.11 (0.11) | .12 | .309 |  |  |  |
| 13-14 | 0.07 (0.12) | .07 | .552 |  |  |  |
| 14-15 | 0.13 (0.09) | .16 | .122 |  |  |  |
| 15-16 | 0.15 (0.12) | .15 | .225 |  |  |  |
| 16-17 | **0.20 (0.09)** | **.21** | **.024** |  |  |  |
| 17-18 | **0.18 (0.08)** | **.17** | **.029** |  |  |  |
| 18-19 | 0.18 (0.10) | .22 | .087 |  |  |  |
| 19-20 | -0.10 (0.11) | -.11 | .340 |  |  |  |
| haq_t_->pib_t+1_ |  |  |  |  |  |  |
| 1-2 | -0.02 (0.09) | -.02 | .841 | -0.11 (0.07) | -.14 | .144 |
| 2-3 | -0.07 (0.11) | -.06 | .541 | -0.11 (0.07) | -.11 | .144 |
| 3-4 | -0.08 (0.11) | -.08 | .443 | -0.11 (0.07) | -.08 | .144 |
| 4-5 | -0.10 (0.11) | -.09 | .399 | -0.11 (0.07) | -.06 | .144 |
| 5-6 | -0.04 (0.13) | -.03 | .777 |  |  |  |
| 6-7 | **0.23 (0.12)** | **.22** | **.043** |  |  |  |
| 7-8 | 0.04 (0.10) | .05 | .671 |  |  |  |
| 8-9 | 0.16 (0.09) | .17 | .075 |  |  |  |
| 9-10 | -0.15 (0.14) | -.13 | .264 |  |  |  |
| 10-11 | -0.03 (0.11) | -.03 | .751 |  |  |  |
| 11-12 | 0.05 (0.13) | .05 | .695 |  |  |  |
| 12-13 | -0.12 (0.13) | -.11 | .356 |  |  |  |
| 13-14 | **0.32 (0.16)** | **.23** | **.046** |  |  |  |
| 14-15 | **0.37 (0.13)** | **.33** | **.004** |  |  |  |
| 15-16 | 0.10 (0.17) | .07 | .570 |  |  |  |
| 16-17 | **0.27 (0.12)** | **.25** | **.028** |  |  |  |
| 17-18 | **0.31 (0.09)** | **.31** | **.028** |  |  |  |
| 18-19 | -0.08 (0.11) | -.09 | .505 |  |  |  |
| 19-20 | 0.18 (0.14) | .16 | .197 |  |  |  |
| r_w_ |  |  |  |  |  |  |
| 1 | -0.01 (0.01) | -.06 | .580 | 0.00 (0.02) | .01 | .993 |
| 2 | 0.01 (0.01) | .06 | .563 | **0.04 (0.02)** | **.31** | **.016** |
| 3 | 0.01 (0.01) | .05 | .657 | 0.03 (0.02) | .35 | .055 |
| 4 | **0.03 (0.01)** | **.24** | **.023** | 0.02 (0.01) | .20 | .242 |
| 5 | 0.02 (0.01) | .16 | .142 | 0.03 (0.02) | .21 | .070 |
| 6 | **0.03 (0.01)** | **.24** | **.032** |  |  |  |
| 7 | **0.05 (0.01)** | **.35** | **.001** |  |  |  |
| 8 | 0.02 (0.01) | .13 | .200 |  |  |  |
| 9 | **0.05 (0.01)** | **.45** | **.000** |  |  |  |
| 10 | 0.03 (0.01) | .20 | .064 |  |  |  |
| 11 | 0.02 (0.01) | .22 | .071 |  |  |  |
| 12 | 0.00 (0.01) | .02 | .891 |  |  |  |
| 13 | **0.03 (0.01)** | **.34** | **.006** |  |  |  |
| 14 | **0.04 (0.01)** | **.36** | **.001** |  |  |  |
| 15 | **0.04 (0.01)** | **.41** | **.000** |  |  |  |
| 16 | **0.03 (0.01)** | **.23** | **.031** |  |  |  |
| 17 | 0.01 (0.01) | .10 | .358 |  |  |  |
| 18 | **0.03 (0.01)** | **.31** | **.007** |  |  |  |
| 19 | **0.04 (0.01)** | **.42** | **.000** |  |  |  |
| 20 | **0.02 (0.01)** | **.23** | **.048** |  |  |  |
| *Between-Person Level* | | | | | | |
| ip_pre_ -> pib | -0.01 (0.08) | -.01 | .902 | -0.03 (0.08) | -.03 | .690 |
| ip_pre_ -> haq | -0.16 (0.10) | -.16 | .085 | -0.18 (0.10)) | -.16 | .067 |
| pib -> ip_post_ | -0.17 (0.11) | -.15 | .103 | -0.21 (0.11) | -.18 | .060 |
| haq -> ip_post_ | -0.17 (0.10) | -.16 | .074 | -0.19 (0.10) | -.18 | .054 |
| iip_pre_ -> ip_post_ | **0.63 (0.09)** | **.55** | **<.001** | **0.61 (0.09)** | **.54** | **<.001** |
| group -> haq | 0.06 (0.09) | .06 | .483 | 0.03 (0.09) | .03 | .705 |
| r_b_ | **0.09 (0.03)** | **.34** | **.001** | **0.10 (0.03)** | **.38** | **.001** |

*Note*. Auto-regressive and cross-lagged effects are constrained. haq = helping alliance questionnaire (therapeutic alliance). PIB = patient interpersonal behavior. Group = study group (competence vs. no feedback). r_b_ = between-person correlation. t = a session at a given time point. t+1 = subsequent session. r_w_ = correlation within sessions.
